# Overview of Silica-Polymer Nanostructures for Waterborne High-Performance Coatings

**DOI:** 10.3390/polym13071003

**Published:** 2021-03-24

**Authors:** Tiago D. Martins, Tânia Ribeiro, José Paulo S. Farinha

**Affiliations:** Centro de Química Estrutural, Department of Chemical Engineering, Instituto Superior Técnico, Universidade de Lisboa, 1049-001 Lisboa, Portugal; tiagodmartins@tecnico.ulisboa.pt (T.D.M.); tania.ribeiro@tecnico.ulisboa.pt (T.R.)

**Keywords:** silica-polymer nanostructures, high performance hybrid films, functional waterborne coatings

## Abstract

Combining organic and inorganic components at a nanoscale is an effective way to obtain high performance coating materials with excellent chemical and physical properties. This review focuses on recent approaches to prepare hybrid nanostructured waterborne coating materials combining the mechanical properties and versatility of silica as the inorganic filler, with the flexural properties and ease of processing of the polymer matrix. We cover silica-polymer coupling agents used to link the organic and inorganic components, the formation of hybrid films from these silica-polymer nanostructures, and their different applications. These hybrid nanostructures can be used to prepare high performance functional coatings with different properties from optical transparency, to resistance to temperature, hydrophobicity, anti-corrosion, resistance to scratch, and antimicrobial activity.

## 1. Introduction

The field of polymer coatings has a huge societal impact [1]. Polymer coatings are used all around us, for the decoration and protection of surfaces, but more importantly to give them different functionalities. Waterborne polymer coatings assume special importance because of their lower environmental impact, due to the use of much lower amounts of volatile organic compounds (VOCs). VOCs are used to facilitate polymer interdiffusion during film formation so as to produce a stronger film upon evaporation. Although the coatings industry have been reducing the use of VOCs [2,3,4], waterborne coatings based on dispersions of polymer nanoparticles (PNPs), also known as latex, do not easily reach the high performance of their solventborne counterparts [5].

### 1.1. Film Formation in Waterborne Coatings

**Figure 1:**
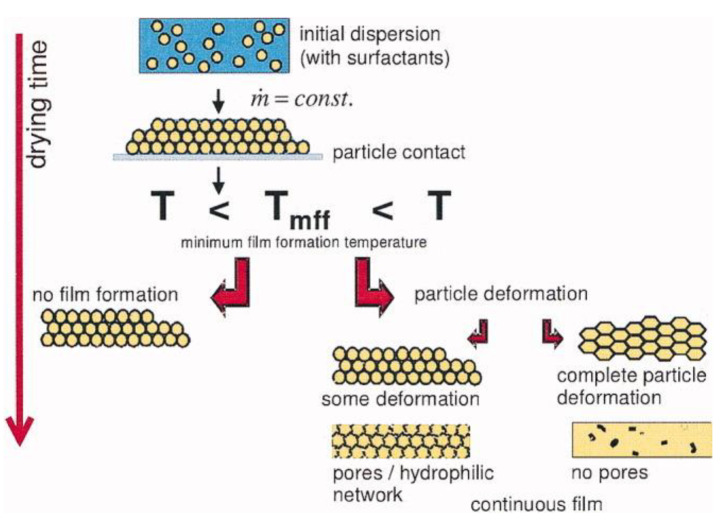
Film formation from water dispersions of polymer nanoparticles (PNPs). Water evaporation leads to a PNP packing, which deform above a minimum film formation temperature (*T_mff_*), as a result of surface tension and capillary forces. Complete particle deformation and chain interdiffusion at *T* > *T_g_* leads to a continuous non-porous film. Reprinted with permission from ref. [8]. Copyright 2007 American Institute of Chemical Engineers (AIChE).

Waterborne polymer coatings are formed in three steps: Evaporation of the solvent (water), deformation of the PNPs, and coalescence by interdiffusion of the polymer chains (Figure 1). Water evaporation leads to a close-packed layer of PNPs and the deformation of the particles from their spherical shape (by a combination of capillary, osmotic, and surface forces), above the “minimum film formation temperature” (*T_mff_*, which is close to the glass transition temperature, *T_g_*, of the polymer in water) [5,6,7,8], to produce a continuous but still mechanically weak film. Coalescence of the nanoparticles by polymer chain interdiffusion across the particle boundaries (above the polymer *T_g_*) produce the final mechanically resistant film [9,10,11]. The mechanical performance of the material depends on the degree of entanglement between polymer chains from across particle boundaries.

Diffusion of polymer chains across nanoparticle boundaries is of paramount importance in the formation of a film with good mechanical properties. The most common techniques to follow diffusion of the polymer chains across the interface between polymer nanoparticles (PNPs) in waterborne films are small angle neutron scattering (SANS) and Förster resonance energy transfer (FRET). SANS experiments can be used to measure the relation between the extent of diffusion and the film tensile strength [12,13], however they require the cumbersome preparation of deuterated and nondeuterated PNPs to obtain the necessary contrast. Measurement of polymer interdiffusion by FRET, on the other hand, requires the labeling of PNPs with appropriate dyes: A fluorescent energy donor and an energy acceptor [14]. FRET can give information on the mixing of polymers labeled with donor and acceptor dyes in a wide range geometries [15,16,17,18], allowing the experimental determination of dye concentration profiles in complex nanostructured materials [19]. FRET has been used to study the effects of different factors on the formation of waterborne coatings, such as curing temperature [20], plasticizers [21], blending [22], crosslinkers [23,24,25], and the presence of filler particles [26,27,28,29].

For these experiments, the films are prepared from a blend of FRET donor- and acceptor-labeled PNPs, for which the fluorescence spectra of the donor overlaps the absorption spectra of the acceptor. Since the molar fraction of the dyes is very low, it is assumed that the donor and acceptor dyes serve only as tracers for the location of the polymer. Since donor and acceptor dyes are located in different particles, FRET measurements can be used to evaluate the extent of mixing during the formation of the polymer film [30,31].

In the process of film formation from waterborne polymer coatings, there is an inherent trade-off between the kinetics of diffusion and the mechanical resistance of the final film. Using polymers with lower molecular weight and/or lower *T_g_* leads to faster polymer interdiffusion, but a final film with lower mechanical resistance. However, using polymers with higher molecular weight and/or higher *T_g_* does not necessarily produce better films because interdiffusion of the polymer chains can be strongly hindered, ultimately producing brittle films.

The traditional approach to this conundrum was to add VOCs as plasticizers to promote faster diffusion by decreasing the *T_g_* of polymer only during film formation. Since the VOCs evaporate from the final film, this allows the use of polymers with a larger molecular weight and higher *T_g_* that result in films with better mechanical resistance. The complete elimination of VOCs from waterborne polymer coating compositions requires new strategies to improve film mechanical resistance. The most promising approaches rely in balancing polymer diffusion with chain crosslinking and incorporating inorganic components in the film, notably silica nanoparticles (SNPs).

### 1.2. Diffusion and Crosslinking

Crosslinking of the diffusing polymer chains during film formation can be used to anchor the chains and thus increase mechanical resistance. Covalent bonding between chains diffusing out of the PNPs in the film form a polymer network that can increase the temperature, solvent, and mechanical resistance of the final films. This three-dimensional network not only reinforces the polymer matrix but also strengthens the interface with other components of the film. However, network formation is only effective if the polymer chains are able to diffuse before the crosslinking reaction takes place, so that film formation depends on the kinetics of both processes [23]. The balance between the diffusion and crosslinking kinetics has been theoretically modeled by De Gennes [6] to explain the role of diffusion and crosslinking on the development of the films and their final properties. The ratio of the diffusion time (*T_diff_*, time for a chain to diffuse out of its initial conformation) and the reaction time (*T_reac_*, time for one cross-link to form in each chain), $\alpha =\;T_{diff}/T_{reac}$, increases when the mobility of the chains is reduced upon crosslinking (*α* >> 1), inhibiting the healing of the interfaces, which results in poor mechanical properties. If crosslinking happens after the significant diffusion of the polymer (*α* << 1), the resulting material will not present mechanical properties better than the bulk material. Careful balancing of crosslinking and interdiffusion (*α* ≈ 1) is necessary to optimize the film’s mechanical properties [5,6,32].

**Figure 2:**
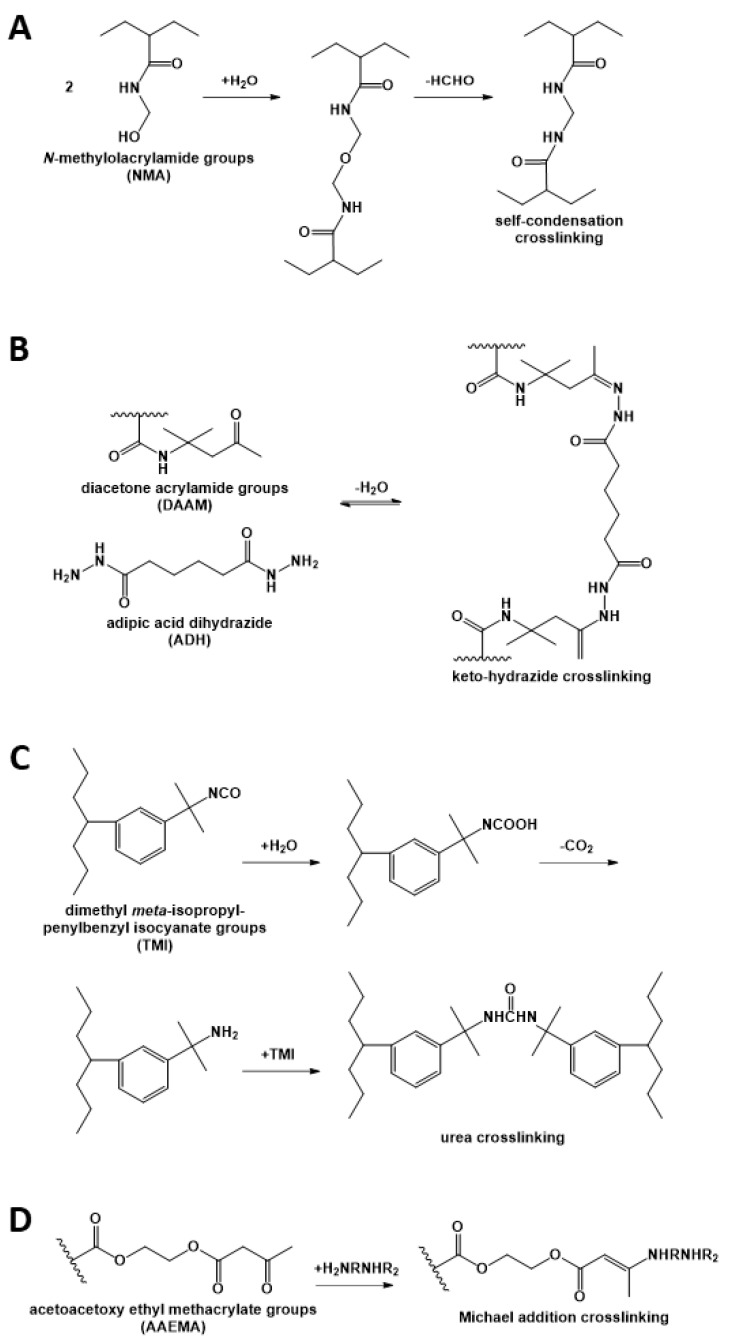
Crosslinking reactions used in waterborne polymer coatings, based on (**A**) *N*-methyloacrylamide; (**B**) diacetone acrylamide (DAAM) and adipic acid dihydrazide; (**C**) dimethyl meta-isopropenylbenzyl isocyanate (TMI); and (**D**) acetoacetoxy ethyl methacrylate (AAEMA) with a diamine.

Crosslinking strategies usually consist in incorporating different reactive groups in different polymer nanoparticles, so that the reaction occurs only upon chain diffusion during film formation, thus preserving the dispersion stability. Different approaches have been used to implement this strategy. For example, dispersions containing *N*-methyloacrylamide or *N*-ethylacrylamide have been studied (Figure 2A), but were found to produce byproducts that are either toxic (formaldehyde) or result in the formation of low molecular weight polymers with weak mechanical properties [7,33,34,35]. On the other hand, the use of the reversible keto-hydrazide crosslinking reaction (Figure 2B) resulted in a small improvement of the mechanical properties of the film because of its low reaction rate, and in addition, hydrazide has toxic effects [36,37,38]. Crosslinking between isocyanate-containing polymers (Figure 2C) resulted in insufficient interfacial crosslinking and thus, lack of mechanical strength [3]. The use of the Michael addition with diamines (Figure 2D) [39] was found to hinder chain diffusion, also yielding polymer films with poor mechanical properties.

### 1.3. Inorganic Nanofillers

A more promising approach to improve the performance of waterborne coatings without compromising the flexural properties of the polymer is the incorporation of nanosized inorganic components (or nanofillers). Although different fillers have been used in coating applications (Table 1) [40,41], silica nanoparticles (SNPs) have received special attention due to their high surface area, cost-effective production, and easy surface functionalization [42]. SNPs can be prepared by simple, scalable, and low-cost techniques and offer tunable and very well-defined size, morphology, and porosity. Hybrid-silica materials combine the rigidity and high thermal stability of the inorganic components with the flexibility, ductility, and processability of the polymer matrix [9]. When used in polymer coating formulations, SNPs can also add new functionalities to the films, providing protection from moisture, temperature, scratching, radiation, and corrosion, while preserving optical transparency and providing specific electrical or mechanical behavior, regulation of microbial adhesion, etc.

In this review, we discuss recent progress in the preparation of high-performance coatings using silica-polymer hybrid nanomaterials, presenting the different silica nanostructures and their functionalization for incorporation into waterborne polymer coating materials. We summarize some of the strategies reported for the incorporation of silica nanostructures in polymeric matrixes in coating applications, and present recent results on functional coating applications based in hybrid waterborne polymer dispersions.

## 2. Preparation and Functionalization of Silica Nanostructures for Coating Applications

Silica nanoparticles (SNP) used in hybrid silica-polymer coatings are usually prepared by flame hydrolysis (fumed silica) or by the Stöber method (colloidal silica). Although fumed silica have a lower cost when compared to colloidal silica, it has a tendency to irreversibly aggregate [9]. Colloidal silica is obtained by the Stöber method, a sol-gel process based on the hydrolysis and condensation of silica precursors, such as tetraethyl orthosilicate (TEOS), in an aqueous medium at a basic pH [57]. High porosity mesoporous silica nanoparticles (MSNs) can be obtained by adding a template to the sol-gel (for example, cetyltrimethylammonium chloride—CTAC, cetyltrimethylammonium bromide—CTAB, or Pluronic F127). MSNs feature tunable diameter and pore size [58,59], and huge surface area and pore volume values that allow extensive and selective surface functionalization, as well as the loading of different cargo [60,61,62,63].

The use of MSNs in hybrid polymer coatings is mostly unexplored, but holds great promise [64,65,66,67,68]. For example, Shi et al. [67] prepared epoxy coatings loaded with MSNs as reservoirs for the corrosion inhibitor 8-hydroxyquinoline. Another study on wear resistance of epoxy coatings, showed that incorporation of MSNs loaded with 2-mercaptobenzothiazole (MBT) in the epoxy coatings improved the micro-hardness and decreased the friction coefficient of the coating, increasing its wear resistance [64]. Liu et al. [66] further showed the preparation of a mesoporous silica coating on graphene oxide nanosheets and its incorporation in styrene-butadiene rubber composites to improve their thermal conductivity.

### 2.1. Compatibilization of Silica Nanostructures with the Polymer Matrix

The incorporation of ca. 10 wt% of silica nanoparticles in polymer coating materials has been described to have the best impact in film properties [9,69,70,71,72]. However, this amount is sufficiently large to often produce significant aggregation of the polar silica nanostructures in the usually hydrophobic polymer matrix, leading to the aggregation of the particles, phase separation, and the formation of a mechanically weak composite.

**Figure 3:**
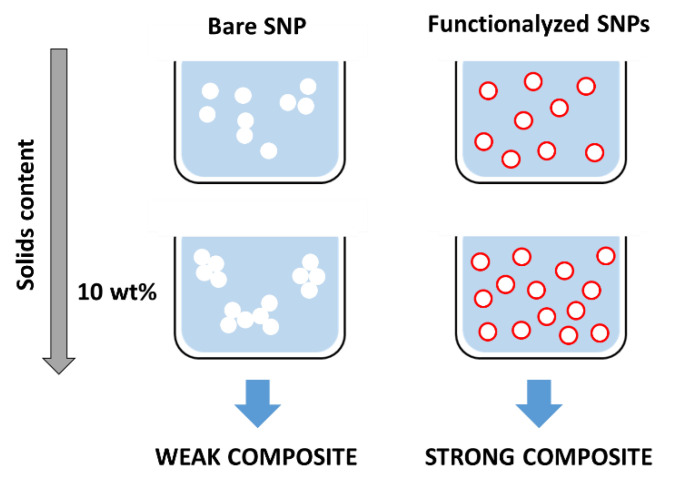
Optimization of hybrid coatings often involve the incorporation of up to ca. 10 wt% of SPNs in polymer material. At such solids content bare SNPs tend to aggregate, resulting in a weak composite (**left**). By appropriately functionalizing the SNPs (**right**), it is possible to obtain homogeneous distributions of the SNPs in the dispersion and polymer matrix, leading to better composite performance.

Since homogeneous films with relatively high solids content are required for high performance waterborne coatings, SNP aggregation must be reduced in order to increase the silica content in the mixture. One strategy to achieve this is by functionalizing the silica surface (Figure 3) to increase the silica-polymer affinity (by promoting van der Waals forces, hydrogen bonds, or ionic interactions), or to covalently link the two components, maximizing the interfacial stability between the silica and polymeric matrix. This can be achieved by functionalizing the silica nanoparticles with a coupling agent that provides grafting of polymer chains onto the SNPs surface. Coupling agents should form siloxane bonds with the SNPs via the hydrolysis and condensation of alkoxy groups, and bear functional groups to connect to the polymer matrix [9,69,70,73].

### 2.2. Silica-Polymer Coupling Agents

Coupling agents not only enhance the compatibility between the organic and inorganic components to obtain homogeneous dispersions and films, but can also avoid cavitation in the films due to the low interaction between the matrix and filler. Coupling agents should thus be chosen according to the polymeric matrix and the material’s desired application. The main criteria for this choice are the ability to bind to the polymer of the matrix (for example, through a polymerizable group), while attaching to the silica network by alkoxysilane groups. Table 2 describes the most common coupling agents used in the preparation of silica-polymer nanostructures. For example, 3-aminopropyl triethoxysilane (APTES) is widely used to bind to epoxy resins [74,75,76] and polyurethane [77], increasing their performance, or even as grafting sites for RAFT agents [78] and ATRP initiators [79]. On the other hand, functionalizing SNPs with γ-(2,3-epoxypropoxy)propyl-trimethoxysilane (GPTMS) can increase the compatibility of the inorganic component within epoxy resins [80,81,82,83,84]. GPTMS can be further modified into carbonate functionalized silanes (4-((3-(trimethoxysilyl)propoxy)methyl)-1,3-dioxolan-2-one, CPS) increasing polyurethane-based coatings performance [85]. ATRP initiators are commercially available as silica precursors, for example 3-(2-Bromoisobutyryl)propyl triethoxysilane (BPTS) [86,87], for directly growing polymer chains in the silica surface.

Another strategy widely used to grow polymer chains from the silica surface is the functionalization with 3-Methacryloxypropyl trimethoxysilane (MPS), which provides anchoring to acrylic monomers, such as butyl methacrylate (BMA) [88,89], butyl acrylate (BA) [72,79,90], methyl methacrylate (MMA) [72,89,90], dodecafluoroheptyl methacrylate (DFMA) [72], bisphenol A-glycidyl methacrylate (Bis-GMA) [91], hexanedioldiacrylate (HDDA) [92], methacrylic acid (MAA) [93], benzyl methacrylate (BnMA), 2-hydroxyethyl methacrylate (HEMA) [94], glycidyl methacrylate (GMA) [95], and vinyl-bearing monomers like styrene (St) [79,96,97]. Increasing the compatibility between silica and styrene has also been performed using vinyltrimethoxysilane (VTMS) [96] or *N*-(3-(trimethoxysilyl)propyl)aniline (PATMS) [98]. Although not used on coating materials yet, triethoxysilylbutyraldehyde (TEBA) can open a new route for covalently bonded hybrid materials [99].

**Table 2:** Most common coupling agents used in the preparation of hybrid silica-polymer materials.

Name		Chemical Structure	Ref.
APTES	3-Aminopropyl triethoxysilane	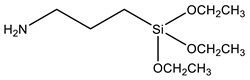	[74,75,76,77,78]
GPTMS	3-Glycidoxypropyl trimethoxysilane	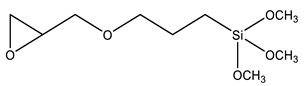	[80,81,82,83,84,100]
CPS	4-((3-(trimethoxysilyl)propoxy) methyl)-1,3-dioxolan-2-one	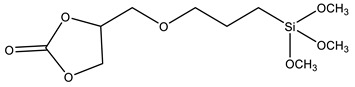	[85]
BPTS	3-(2-Bromoisobutyryl)propyl triethoxysilane	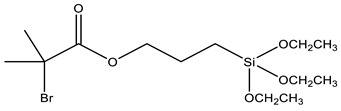	[86,87]
MPS	3-Methacryloxypropyl trimethoxysilane	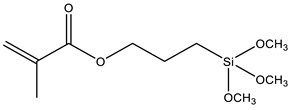	[29,72,79,88,89,90,91,92,93,94,95,96,97]
VTMS	Vinyltrimethoxysilane	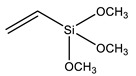	[96]
PATMS	*N*-[3-(trimethoxysilyl)propyl]aniline	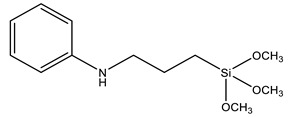	[98]
TEBA	Triethoxysilylbutyraldehyde	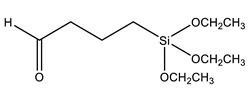	[99]
PFPS	Pentafluorophenyltriethoxysilane	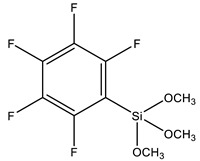	[101]

The effect of the coupling agent on the surface of SNPs is critical for specific properties of the polymer-silica nanostructure, such as its hydrophilicity, hydrophobicity, or chemical binding ability [5,73]. Zhi et al. [84] have functionalized the surface of the SNPs with GPTMS for better compatibility with epoxy resin. They were able to achieve strong bonds between the silica and polymer matrix, obtaining a nanometer-scale surface roughness that resulted in a superhydrophobic material. Jouyandeh et al. [100] worked on the functionalization of nanoparticles with nitrogen-rich macromolecules that would drive crosslinking reactions with pyromellitic acid dianhydride. In this case GPTMS was used to firstly bind the super reactive hyperbranched polyethylenimine (PEI) that would later be grafted on the silica surface, significantly improving the performance of the material. Xu et al. [101] functionalized SNPs with pentafluorophenyltriethoxysilane (PFPS), enabling its dispersion within hyperbranched fluoropolymer (HBFP). The chemically modified silica nanoparticles presented reactive functionalities that were later covalently integrated into the complex networks.

A completely different approach to enhance the compatibility within the hybrid material was developed by Kumar et al., who modified the SNPs surface with ^60^Co-gamma radiation to induce the grafting of GMA and HEMA. This effectively increased the compatibility of the SNP with the vinyl polymeric matrix, increasing the performance of the coating [102].

## 3. Incorporation of Silica in Polymer Materials

**Figure 4:**
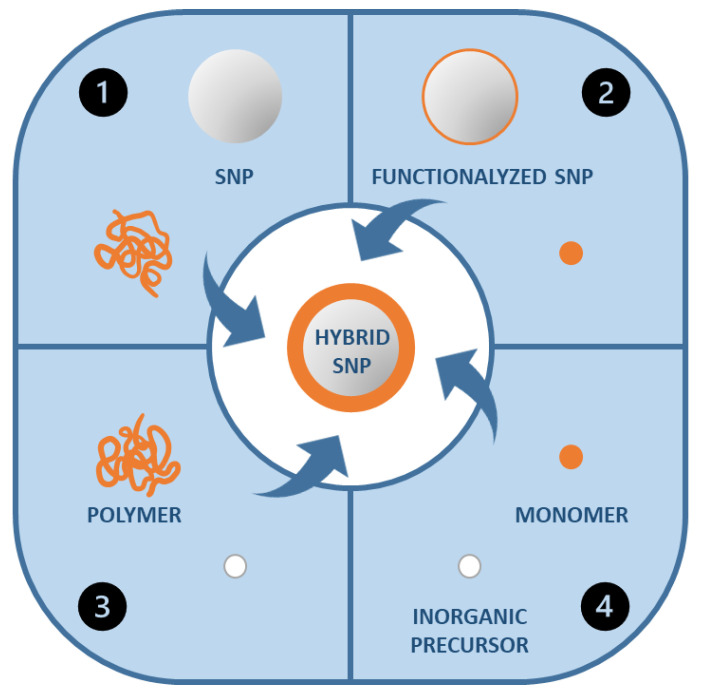
Different synthetic strategies for the formation of polymer-silica hybrid nanoparticles.

The more common methods to prepare hybrid organic/inorganic coating materials are (Figure 4): (1) The combination of the polymer chains or polymer nanoparticles with the inorganic nanoparticles; (2) the polymerization of the organic component in the presence of inorganic nanoparticles; (3) the formation of the inorganic component in the presence of the polymer chains or nanoparticles; and (4) the simultaneous formation of both polymer and inorganic components [103].

**Figure 5:**
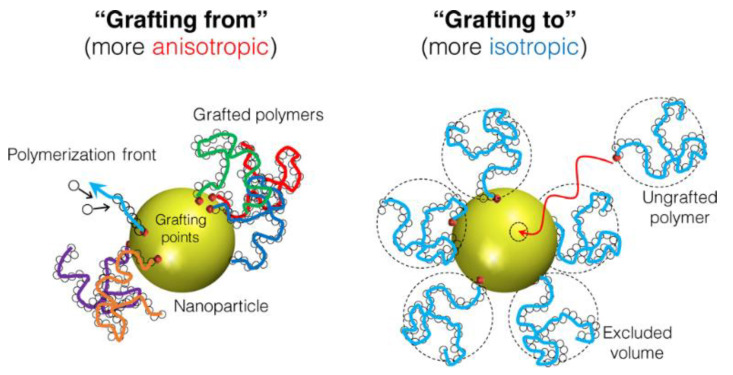
Surface coverage by “grafting from” and “grafting to” approaches. Reprinted with permission from ref. [106]. Copyright 2017 ACS.

While (1) can lead to the aggregation of the inorganic component and phase separations as discussed above, and (3) and (4) do not offer general processing advantages, strategy (2) allows the preparation of very homogeneous hybrid polymer films. This approach can involve emulsion polymerization in the presence of inorganic nanoparticles [29,88] or the modification of surface functionalized inorganic particles with polymer chains (see above) to promote their homogeneous dispersion in the matrix. In the last case, the polymer can be “grafted from” the surface of the inorganic nanoparticles [79,104] or “grafted to” the nanoparticles [79] (Figure 5) among other less used possibilities [105]. In the “grafting from” approach, the polymer chains grow from reactive groups on the surface of the inorganic nanoparticles. In the “grafting to” approach, the polymer chains are previously formed in solution and covalently bonded to the surface of the inorganic nanoparticles [103,106]. The “grafting from” method is generally believed to produce higher polymer density, but “grafting to” allows a more uniform coverage of the surface.

In “grafting from” it is possible to use conventional free radical polymerization (on SNPs surface-modified with monomer units), or a controlled radical polymerization technique to control the composition, molecular weight, and molecular weight dispersity of the chains [107,108]. Atom transfer radical polymerization (ATRP) and reversible addition–fragmentation chain transfer (RAFT), are the most used techniques for a precise design of the polymer chains. While in ATRP, the SNP surface is modified with the initiator [79,109], in RAFT the surface of the SNPs is modified with a chain transfer agent (CTA) [61,97,110,111].

**Figure 6:**
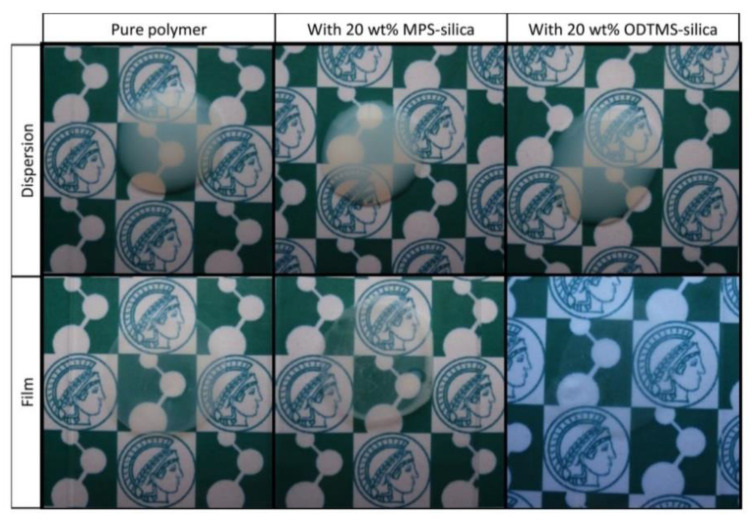
Dispersions (**top**) and the corresponding films annealing at 100 °C for 24 h (**bottom**) obtained using BMA:MMA (butyl methacrylate:methyl methacrylate) nanoparticles without silica (pure polymer), with 20 wt% of silica nanoparticles (SNPs) modified with MPS, and with 20 wt% of SNPs modified with ODTMS (N-octadecyltrimethoxysilane). Reprinted with permission from ref. [89]. Copyright 2016 John Wiley and Sons.

Another approach involves encapsulation of the SNP by emulsion polymerization. In this case, the SNPs are usually surface-modified with monomer units so that these can be used as seeding particles in the emulsion/miniemulsion polymerization, effectively reducing SNP aggregation in the final coatings (Figure 6) [29,74,75].

**Figure 7:**
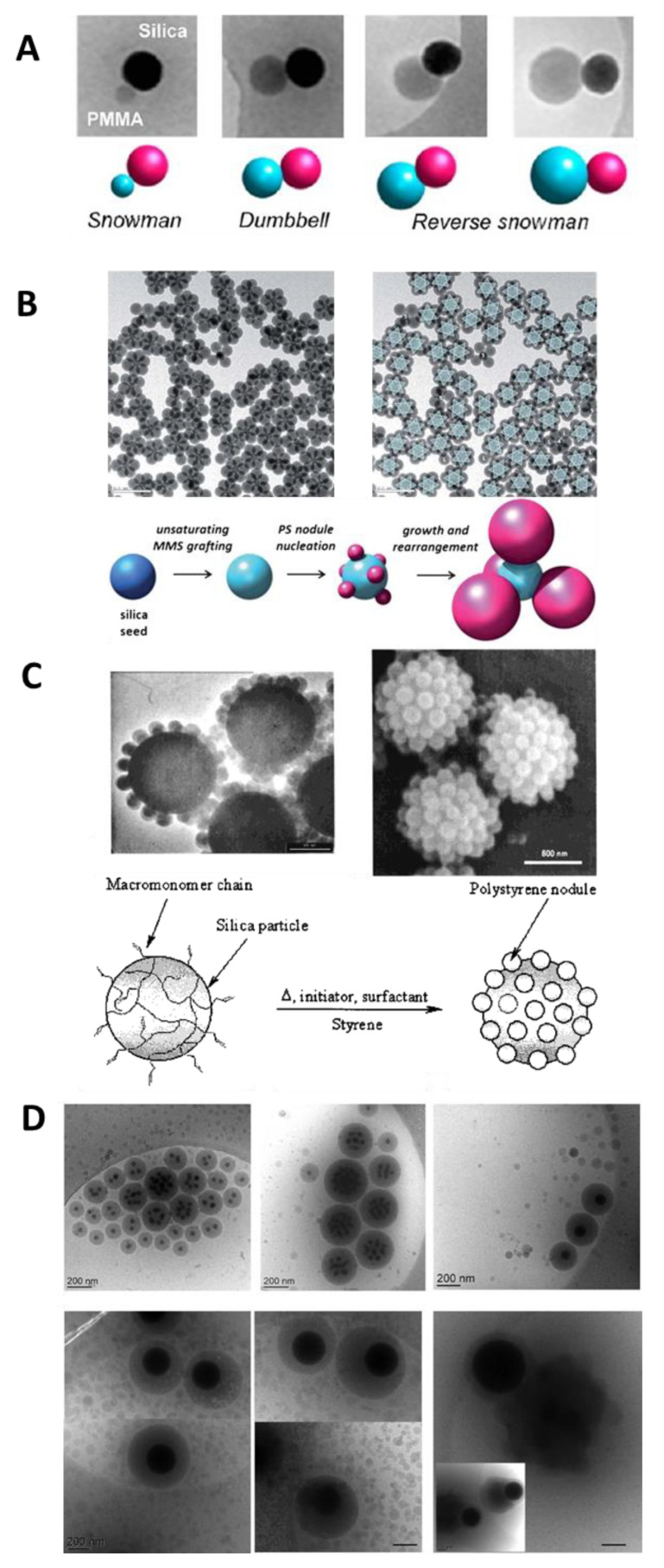
Hybrid particle morphology. (**A**) Dissymmetrical snowman- and dumbbell-like silica/polymer colloidal particles through emulsion polymerization of MMA or St using bicationic initiator previously anchored on the silica surface. Reprinted with permission from ref. [113]. Copyright 2012 ACS. (**B**) Hexapods obtained by St emulsion polymerization. Reprinted with permission from ref. [112]. Copyright 2012 The Royal Society of Chemistry. (**C**) Raspberry-like hybrids based on 1-µm silica particles. Reprinted with permission from ref. [114]. Copyright 2002 ACS. (**D**) Encapsulated SNPs showing the effect of the SNPs size on the morphology of the hybrid particles (scale bar: 200 nm). Reprinted with permission from ref. [116]. Copyright 2016 ACS.

The work of Désert et al. [112] reports the controlled morphology of the seeded emulsion polymerization of styrene on functionalized SNPs presenting a cluster morphology. The silica concentration and the feeding process have a strong impact on the particle morphology, yielding cluster-like [112], snowman-like [113], raspberry-like [95,114,115], or encapsulated core-shell hybrid nanoparticles [103,104,105] (Figure 7). Among the different morphologies, some have had no application in hybrid coatings (i.e., cluster-like and snowman-like nanoparticles). However, the morphology of the nanoparticles can impact the properties to the coating as described for the raspberry structures that lead to superhydrophobic properties [84]. Nanoparticles with a core-shell morphology, usually provide the more homogeneous dispersion in the polymer matrix [116].

**Figure 8:**
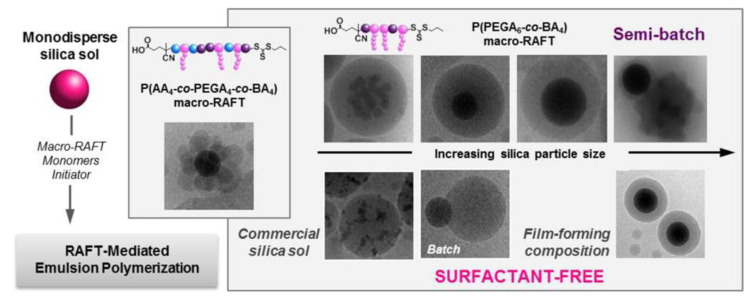
Illustration for the formation processes hybrid latexes by surfactant-free RAFT-mediated emulsion polymerization. Reprinted with permission from ref. [116]. Copyright 2016 ACS.

The surfactants used in emulsion polymerization can however have negative effects on the final properties and appearance of the coatings. Examples of surfactant-free emulsion polymerization for the encapsulation of SNPs include the combination of RAFT with emulsion polymerization, using a macro-RAFT agent that adsorbs onto the surface of the SNPs (Figure 8) [116] and the use of ionic comonomers (the sodium salt of styrene sulfonic acid and potassium methacrylate) to stabilize SNPs functionalized with MPS, to which poly(MMA-co-BA) was grafted [90].

## 4. Applications of Hybrid Nanostructured Films

Hybrid nanoparticles offer huge design flexibility for the design of coatings with a wide range of properties, improving not only the mechanical properties (e.g., impact, abrasion, or scratch) and chemical resistance (e.g., against oxidation and hydrolysis resulting from exposure to sunlight, air, and water), but also providing new functionalities (Table 3) [4,9]. Desirable properties in waterborne coatings include flexibility [29,88,117], adhesion [81,118,119], wear resistance [92,102,120,121,122], and durability [120]. Among the possibilities for added coating functionality are, for example, flame retardancy [119], solvent and chemical resistance [123,124], stain resistance [102,124], anti-cavitation [81], extreme robustness (for space-based applications) [125], antimicrobial activity [126,127,128,129,130,131], superhydrophobicity [84,95,124,132,133,134,135,136,137,138], or photoactive fluorescent coatings [29] presented in Table 3.

Often, the use of SNPs can improve different coating properties and simultaneously add new functionalities. For example, SNPs increase the *T_g_* and hardness of polyurethane composite films, containing ethylene glycol methacrylate phosphate (EGMP), with the resulting material presenting also good adhesion to steel surfaces and flame retardancy [119]. In fact, silica-containing epoxy composites are already used in adhesives, paints, solvent and chemical resistance, and marine coating technology [118,123,130]. The use of 3-glycidyloxypropyl trimethoxysilane (GPTMS) and TEOS on epoxy composites lead to coating films with improved break resistance due to the hyperbranched structure of GPTMS, but also increased thermal stability and erosion resistance, for anti-cavitation coating applications [81]. SNPs modified with toluene diisocyanate (TDI) groups were used in high performance phenylene sulphide (PPS) nanocomposite coatings showing increased tensile strength and hardness [121].

Although the introduction of SNPs can increase the mechanical properties of coating materials, SNPs can also show negative effects on the final coatings. For example, they can impact crosslinking reactions in the films and their curing kinetics. For example, the addition of SNPs to water-based alkyd coatings changed their autoxidation curing kinetics, depending on the morphology of the nanoparticles and their aggregation level [139].

Different functionalities can be imparted to hybrid nanocomposite coatings by using SNPs. For example, even though SNP are non-toxic (they are usually considered biocompatible and are endogenous to most living organisms), they have been used to be used to impart antibacterial properties to coatings by taking advantage of their ease of processability and surface functionalization to carry antibacterial agents. Yamashita et al. [131] described a silicone rubber coating with antimicrobial activity against *S. aureus* and *E. coli* containing hybrid nanoparticles with a silica core and a poly(p-styrene tributyltetradecylphosphonium sulfate) shell. Silicone rubber containing SNPs grafted with poly(vinylbenzyltributylphosphonium chloride) also show antibacterial activity against *S. aureus*, *E. coli*, and *P. aeruginosa* [129]. The antibacterial activity is strong even at very low nanoparticle concentrations (0.1 wt%) in different formulations (silicone rubber, polystyrene, and commercial paints). Hybrid nanoparticles obtained by co-condensation of *N*-(3-triethoxysilylpropyl)-5,5-dimethylhydantoin and TEOS, followed by chlorination, showed excellent antimicrobial activities against *E. coli* and *S. aureus*, with good storage stability for application in coating materials [127]. Antibacterial behavior against the same strains was also obtained for coating formulations containing polyols from Linseed and Castor oils as the organic fillers and TEOS as the inorganic constituent [128]. The chemical structure of this biodegradable family of materials can suffer chemical transformations yielding low molecular weight polymeric materials useful for eco-friendly coatings [140,141].

**Figure 9:**
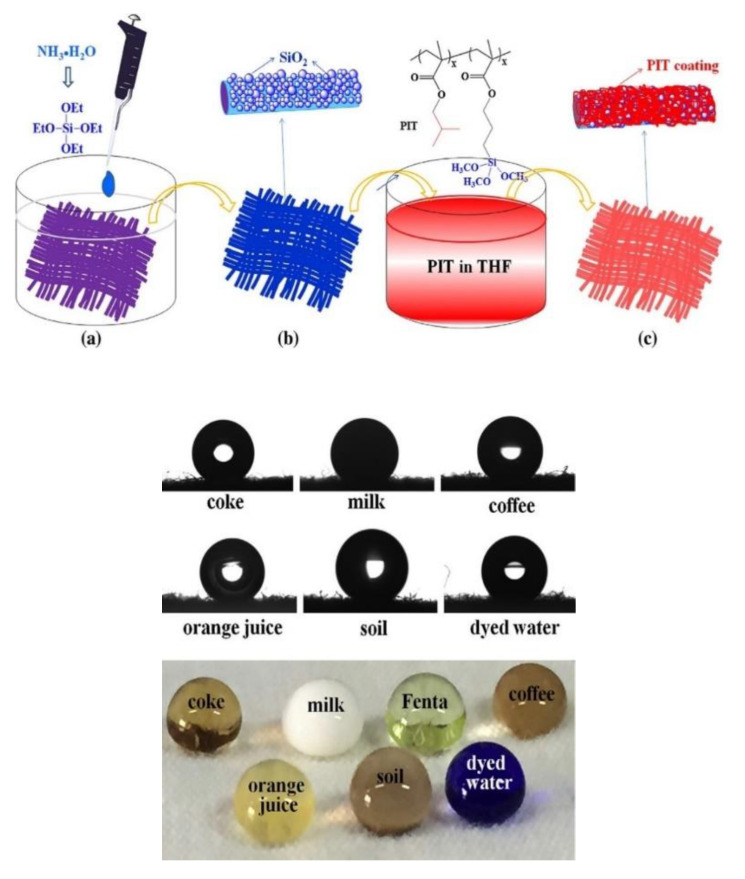
Hydrophobization of fabric with silica and polymeric water-repellent PIT. (**a**) Alkali-treated polyester fabric, (**b**) SiO_2_@fabric, and (**c**) PIT hydrophobized SiO_2_@fabric. The anti-stain properties of the fabric are observed upon trial with several aqueous mixtures (**bottom**). Reprinted with permission from ref. [124]. Copyright 2017 Elsevier.

Nano-structuring of the coating film surface by the SNP has been explored to impart superhydrophobicity to coatings. This is an extremely interesting property, with application in self-cleaning, anticorrosion, antipollution, self-healing, and ice repellent surfaces [142]. Coatings with contact angles above 150° have been prepared using nanoparticles modified with different hydrophobic groups (e.g., fluorinated) [124,133,134,135,136,138] and modulating the roughness of the surface (e.g., using raspberry-like structures) [95,115,132]. Nahum et al. prepared nanocomposite coatings based on urethane acrylate and epoxy, containing SNPs functionalized with photoreactive benzophenone groups and a fluorinated silane [134]. These coatings are UV-cured to yield high contact angles and a good bonding between the SNPs and polymeric matrix. Water-repellent fluorine-free coatings were also reported, based on nanostructured roughness provided by the incorporation of SNP in polyester [124]. After treatment with poly(isobutylmethacrylate-co-3-methacryloxypropyltri-methoxysilane) (PIT), hydrophobic polyester fabrics with good water repellence were obtained (Figure 9).

**Figure 10:**
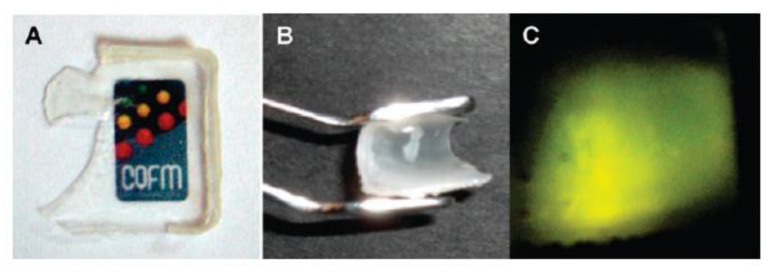
Hybrid film showing transparency (**A**), flexibility (**B**), and strong fluorescence emission under excitation at λ_exc_ = 450 nm (**C**). Reprinted with permission from ref. [88]. Copyright 2009 ACS.

SNPs not only feature high chemical and mechanical stability, but also have the ability to protect guest molecules incorporated in the silica structure. They offer an excellent support for photoactive molecules since they are transparent in a wide range of wavelengths, from the ultraviolet to the near infrared (NIR), and they shield the dyes from oxygen, enhancing their photostability [29]. For example, fluorescent waterborne coatings were prepared from nanoparticles with a silica core and a polymer shell that originates from the coating film [88]. The SNPs were modified by the incorporation of a perylene diimide (PDI) derivative covalently linked to the silica network during the synthesis of the SNPs. The silica shields the dyes from oxygen, increasing their photostability, while the anchoring groups reduce dye aggregation and consequent fluorescence quenching. The SNPs surface was modified with MPS to graft a poly(butyl methacrylate) shell by emulsion polymerization. The hybrid particles yield films with good flexibility and transparency, strong fluorescence emission, and high mechanical strength (Figure 10). Compared to the films without the SNPs, those with hybrid nanoparticles have improved mechanical strength and higher *T_g_* due to the lower polymer mobility [29,143].

## 5. Conclusions and Outlook

The need to replace solvent-borne coatings by environmentally friendly waterborne coatings without losing performance has led to the development of different hybrid coating solutions. Those containing silica nanoparticles (SNP) offer the opportunity to design coatings with good mechanical performance and also other properties, such as super-hydrophobicity, anti-corrosion, antimicrobial activity, optical activity, etc.

The combination of polymer and SNPs can improve the mechanical properties and the chemical resistance of hybrid coatings, but also offers the flexibility to develop high-performance environmentally friendly coatings with a wide range of functions. In these types of materials, the properties and performance are highly dependent on the silica content and the homogeneity of nanoparticles distribution in the coating film. Therefore, coupling agents play a major role in the nanofiller incorporation. These agents contain both reactive groups to attach to the polymer component and to anchor to the SNPs. This approach is particularly interesting in obtaining core-shell nanostructures, with a silica core and a polymer shell, offering excellent control over the distribution of the inorganic component in the polymer matrix of the coating, providing better mechanical performance and avoiding cavitation. The versatility of the preparation and surface modification of SNPs provide the design flexibility to use them as vehicles to impart different functionalities for specific coating applications, from flame retardancy to antimicrobial properties or photoactivity. Other functions, based on the structuring of the coating film provided by the nanoparticles have attracted strong interest, namely to produce anti-stain and superhydrophobic coatings. Better control of the nano-structuring in hybrid coatings offer other still underexplored possibilities, such as the development of structural color.

Silica-polymer hybrid nanostructures still hold untapped potential for the development of high performance environmentally friendly functional coatings for a plethora of novel applications.

## Figures and Tables

**Table 1:** Inorganic fillers other than silica nanoparticles (SNPs) used in hybrid materials for coating applications.

Inorganic Filler	Organic Matrix	Properties	References
Clay	Polyimide	Adhesive strength, abrasion resistance, impact strength, water absorption resistance	[43,44]
	Epoxy	Abrasion resistance, water vapor barrier, corrosion resistance	[45,46]
Carbon Nanotubes	Epoxy	Tensile strength, electric insulation	[47,48]
Graphene Oxide	Poly(vinyl butyral)	Corrosion resistance, superhydrophobicity	[49]
	Epoxy	Corrosion resistance, superhydrophobicity	[50,51,52,53]
Zeolite	Epoxy	Corrosion resistance	[54]
TiO_2_	Metal-quinoline derivatives; poly(methyl methacrylate)	Corrosion resistance, low friction	[55,56]

**Table 3:** Examples of hybrid silica-polymer coating applications.

Applications	Organic Matrix	References
Flame retardancy	Polyurethane, EGMP ^a^	[119]
Solvent and chemical resistance	Epoxy	[123,124]
Stain resistance	Epoxy	[102]
	PIT ^b^	[124]
Anti-cavitation	Epoxy	[81]
Robustness	Phenylene Sulfide	[121]
	Nylon-6	[125]
Antimicrobial	PQDMAEMA ^c^, PTMOSPMA ^d^	[126]
	TS-DMH ^e^	[127]
	Polyols	[128]
	PVBBPC ^f^, Silicone rubber	[129]
	Epoxy	[130]
	PSBDPS ^g^	[131]
Superhydrophobic	Epoxy	[84]
	Epoxy-functionalized methacrylate	[95]
	PIT ^b^	[124]
	TSI-PDMAEMA-PS ^h^	[132]
	Fluoroloakylosiloxane polymer	[133]
	Urethane acrylate	[134,135]
	PFCP ^i^ -based chlorosilane	[136]
	DFMA ^j^	[137]
	Poly(methyl hydrosiloxane)	[138]
Photoactive, fluorescent	Poly(butyl methacrylate)	[29]

^a^ Ethylene glycol methacrylate phosphate; ^b^ poly(isobutylmethacrylate-co-3-methacryloxypropyltrimethoxysilane); ^c^ poly(2-(dimethylamino)ethyl methacrylate); ^d^ poly-(3-(trimethoxysilyl)propyl methacrylate); ^e^ *N*-(3-triethoxysilylpropyl)-5,5-dimethylhy-dantoin; ^f^ poly(vinylbenzyltributylphosphonium chloride); ^g^ poly(p-styrene tributyltetradecylphosphonium sulfate); ^h^ trimethoxysilane-end-capped poly(dimethylaminoethyl methacrylate)-block-polystyrene; ^i^ perfluorocyclopentene; ^j^ dodecafluoroheptyl methacrylate.
